# Shiga toxin type-2 (Stx2) induces glutamate release via phosphoinositide 3-kinase (PI3K) pathway in murine neurons

**DOI:** 10.3389/fnmol.2015.00030

**Published:** 2015-07-14

**Authors:** Fumiko Obata, Lauren M. Hippler, Progyaparamita Saha, Dakshina M. Jandhyala, Olga S. Latinovic

**Affiliations:** ^1^Department of Microbiology and Immunology, University of Maryland School of MedicineBaltimore, MD, USA; ^2^Department of Molecular Pathology, Graduate Faculty of Interdisciplinary of Research, Graduate School, University of YamanashiChuo, Japan; ^3^Department of Molecular Biology and Microbiology, Tufts UniversityBoston, MA, USA; ^4^Institute of Human Virology, University of Maryland School of MedicineBaltimore, MD, USA

**Keywords:** Shiga toxin, *Escherichia coli*, glutamates, PI3K, neurons

## Abstract

Shiga toxin-producing *Escherichia coli* (STEC) can cause central nervous system (CNS) damage resulting in paralysis, seizures, and coma. The key STEC virulence factors associated with systemic illness resulting in CNS impairment are Shiga toxins (Stx). While neurons express the Stx receptor globotriaosylceramide (Gb_3_) *in vivo*, direct toxicity to neurons by Stx has not been studied. We used murine neonatal neuron cultures to study the interaction of Shiga toxin type 2 (Stx2) with cell surface expressed Gb_3_. Single molecule imaging three dimensional STochastic Optical Reconstruction Microscopy—Total Internal Reflection Fluorescence (3D STORM-TIRF) allowed visualization and quantification of Stx2-Gb_3_ interactions. Furthermore, we demonstrate that Stx2 increases neuronal cytosolic Ca^2+^, and NMDA-receptor inhibition blocks Stx2-induced Ca^2+^ influx, suggesting that Stx2-mediates glutamate release. Phosphoinositide 3-kinase (PI3K)-specific inhibition by Wortmannin reduces Stx2-induced intracellular Ca^2+^ indicating that the PI3K signaling pathway may be involved in Stx2-associated glutamate release, and that these pathways may contribute to CNS impairment associated with STEC infection.

## Introduction

Shiga toxin-producing *Escherichia coli* (STEC) infection is an important cause of food poisoning, often associated with contaminated beef and leafy vegetables. STEC associated disease can include diarrhea, bloody diarrhea, hemolytic uremic syndrome (HUS), as well as central nervous system (CNS) impairment. More than 175,000 people per year in the USA are estimated to be infected by STEC (Scallan et al., 2011), and studies have shown the frequency of CNS manifestation to be as high as 42% among STEC-HUS patients (Cimolai et al., 1992; Siegler et al., 1994; Verweyen et al., 1999; Gerber et al., 2002; Oakes et al., 2006; Iijima et al., 2008), such as cortical blindness, poor fine-motor coordination, seizures, and changes in consciousness including coma, indicating a broad spectrum CNS impact. Also, CNS manifestations are often associated with mortality or severe sequelae with the mortality rate of HUS patients showing CNS signs/symptoms being two- to three-fold higher than that of HUS alone (Bale et al., 1980; Upadhyaya et al., 1980; Brasher and Siegler, 1981; Sheth et al., 1986; Cimolai et al., 1992; Siegler, 1994; Siegler et al., 1994). Importantly, CNS symptoms that appear prior to or in absence of HUS have been reported following STEC infection (Bale et al., 1980; Upadhyaya et al., 1980; Brasher and Siegler, 1981; Taylor et al., 1986; Siegler, 1994). In 2011, a European outbreak of STEC resulted in more than 4000 infections with the highest recorded percentage of HUS (22%) (Werber et al., 2013). Furthermore, the outbreak was associated with rather frequent and severe levels of neurological symptoms. To illustrate this, in one cohort, 104 of 217 patients with complicated STEC infection developed neurological manifestations (Magnus et al., 2012). This outbreak was caused by a unique STEC strain with enteroaggregative properties but with the ability to express *stx2* (Jandhyala et al., 2013). This outbreak alerts us both to the potential impact of new emerging infectious diseases, as well as to the importance of STEC-associated CNS disease. The mechanism(s) by which Stx2 promotes neurological symptoms is poorly understood. To date, no specific therapy is available for prevention or remediation of STEC-associated CNS complications emphasizing the need for a better understanding of Stx2-associated CNS impairment.

Various Stx2-associated animal models with CNS symptoms have been reported, including those using pigs, rabbits, rats, or mice (Tzipori et al., 1988; Fujii et al., 1996; Mizuguchi et al., 1996; Sugatani et al., 2000; Garcia et al., 2008; Obata et al., 2008; Takahashi et al., 2008; Tironi-Farinati et al., 2010). Among them, the most common neurological symptom is hindlimb paralysis (Tzipori et al., 1988; Fujii et al., 1996; Mizuguchi et al., 1996; Sugatani et al., 2000; Garcia et al., 2008; Obata et al., 2008; Takahashi et al., 2008; Tironi-Farinati et al., 2010; Obata and Obrig, 2014). In mice, unilateral hindlimb paralysis is seen after Stx2 injection (Obata et al., 2008) that partially resembles to hemiparesis and hemiplegia of STEC-patients. Moreover, we reported that both human and mouse CNS neurons display the Stx2 receptor globotriaosyl ceramide (Gb_3_) and Stx2 and Gb_3_ can be detected in lumbar spinal cord motor neurons in Stx2-injected mice (Obata et al., 2008). However, the direct interaction of Stx2 with neurons and the effect of the toxin on neurons have not been investigated *in vitro* in any mechanistic detail. In this article, we used murine postnatal neuronal cultures to investigate the binding of Stx2 via receptor Gb_3_ expressed on the surface of the cells. Moreover, we determined that Stx2 induces glutamate release via a PI3K-involved signaling pathway in cultured neurons.

## Materials and methods

### Animals

Timed pregnant female C57BL/6 and 4–6 week old male C57BL/6 mice were purchased from Charles River laboratories (Wilmington, MA, USA). Mice were housed with a 12/12 h light and dark cycle and given access to food and water *ad libitum* until the birth of neonates or the end of the experiments. On the day of birth, the mother is separated from neonates and euthanized. For *in vivo* studies of adult mice, male mice were injected intraperitoneally with 400 ng/kg Stx2. To harvest CNS tissues, mice were euthanized with CO_2_ inhalation and perfused with saline from the heart, followed by 4% paraformaldehyde (Sigma-Aldrich, St. Louis, MO) in phosphate buffered saline (HyClone/ThermoFisher Scientific, Waltham, MA) (4% PFA/PBS). Brains and spinal cords were harvested. All animal procedures were performed under an animal use protocol that was reviewed and approved by the Institutional Animal Care and Use Committee at University of Maryland School of Medicine Office of Animal Welfare Assurance (IACUC protocol #0811012). The animal use protocol complies with the Animal Welfare Act, Public Health Service Policy on Humane Care and Use of Laboratory Animals and the Guide for the Care and Use of Laboratory Animals.

### Cell cultures

The hippocampal neuronal cultures were made from post natal day 0 (P0) mouse brain using neurobasal-A media without phenol red (Gibco/Life Technologies, Grand Island, NY) supplemented with 0.5 mM L-glutamine, 50 U/ml penicillin, 50 μg/ml streptomycin, 1X B27 according to the manufacture's instruction. Cells were seeded on poly-L-lysine (Sigma-Aldrich)-coated glass bottom petri dishes (MatTek, Ashland, MA) at 1 × 10^4^ cells per dish for imaging experiments and on lysine-coated 100 mm diameter plastic dishes at 2 × 10^6^ cells per dish for lysate experiments. Day *in vitro* 1 (DIV1) is defined as the day the tissue culture was started. On DIV4 a half of the culture volume was replaced with fresh culture media. DIV7 cultures were used for experiments. Vero cells were purchased from American Type Culture Collection (ATCC, Manassas, VA) and maintained in RPMI1640 media (Gibco/Life Technologies) supplemented with 10% fetal bovine serum (HyClone/ThermoFisher Scientific), 1 mM L-glutamine (Cellgro/Mdiatech, Manassas, VA) and 100 U/ml penicillin and 100 μg/ml streptomycin (Gibco/Life Technologies).

### Purification of Stx2 with LPS removal

Purification of Stx2 was kindly provided by Dr. Anne Kane (The Phoenix Lab at Tufts Medical Center, Boston, MA) (Donohue-Rolfe et al., 1989; Stone et al., 2012). LPS was removed from purified Stx2 by Detoxi-gel from Thermo Fisher Scientific (Rockford, IL, USA) as per the manufacturer's protocol. A phosphate-buffered saline (PBS)-eluted and 0.2 μm filter sterilized Stx2 fraction was tested for LPS [<0.03 endotoxin unit (EU)/ml] using the Limulus amebocyte lysate (LAL) assay (Pyrotell, Associates of Cape Cod Incorporated, East Falmouth, MA, USA). Stx2 fraction was LPS negative in this LAL assay.

### Immunofluorescence of Stx2-treated cells

Cells were incubated with 20 nM Stx2 at 37°C for 5 or 15 min, or with medium alone (0 nM Stx2 control). At the end of the incubation, dishes were placed on ice to stop the internalization of Stx2 and washed with ice cold PBS, for 5 min. Cells were fixed with 4% PFA/PBS, washed and blocked with 3% bovine serum albumin (BSA, Sigma-Aldrich) in PBS. Cells were not permeabilized in order to promote detection of antigen on the cell surface. Cells were treated with anti-Gb_3_ rat monoclonal antibody (clone 38-13, Immunotec/Beckman Coulter, Brea, CA) diluted (1:100) in 1% BSA/PBS incubation at 4°C for overnight, followed by washing in PBS and adding anti-rat IgM-AlexaFluor488 (Life Technologies) diluted (1:2000) in PBS and incubated at room temperature for 2 h. After washing, cells were incubated with anti-Stx2A mouse monoclonal antibody (11E10, ATCC) diluted in 1% BSA/PBS at 4°C for overnight, followed by washing in PBS and treating with anti-mouse IgG-AlexaFluor647 (Life Technologies) at room temperature for 2 h. Cells were washed again in PBS and nuclei were stained with 4′,6-diamidino-2-phenylindole (DAPI, Life Technologies). Duplicate samples were prepared, one for confocal microscopy and the other for three dimensional STochastic Optical Reconstruction Microscopy—Total Internal Reflection Fluorescence microscope (3D STORM-TIRF) observation. Samples were kept in PBS at 4°C until microscopic observation.

### Confocal microscopy

Confocal microscopy was performed using a LSM510 (Carl Zeiss, Thornwood, NY) with Argon (488 nm, 25 mW), Helium Neon (633 nm, 6 mW) and Mai Tai (710 nm, 2 photon excitation for DAPI, 1.5 W) lasers to visualize AlexaFluor-488, -647, and DAPI, respectively. In addition, differential interference contrast microscopy (DIC) was also performed. Image acquisition and analysis were performed using the LSM 5 Image Browser. Confocal experiments were performed using the confocal microscopy facility at University of Maryland School of Medicine.

### Three dimensional STORM-TIRF

Buffer of the stained cells was changed from PBS to STORM-imaging buffer [50 mM Tris-HCl (pH 8.0), 10 mM NaCl, 10% glucose, 5.6 mg/ml glucose oxidase, 0.17 mg/ml catalase, 100 mM MEA (cysteamine), all from Sigma-Aldrich] right before the imaging and kept in the same buffer during the imaging. 256 × 256 pixel fields comprising approximately one cell per field were imaged individually. A TIRF degree of 2660 was used with the perfect focus 3D STORM setting upon sample bleaching for the certain angles just below and above of the indicated TIRF angle of interest. The sample bleaching eliminates non-specific signals and provides the true signals of the designated fluorophores. Argon (488 nm) and Helium Neon (647 nm) lasers were used at 100% power at acquisition in order to activate all possible fluorescence molecules and provide the maximum for the specific signal of interest. A total of 10,000 events were collected per image. Nikon NIS-Elements software was used to acquire and analyze collected STORM-TIRF images. The diameter of one fluorescence molecule was 10 nm. Twelve 488 (Gb_3_)/647 (Stx2) double positive clusters were measured for the size in molecules. The 488 cluster size was calculated as 39.25 ± 14.97 fluorescence molecules per cluster and the 647 cluster size was calculated as 42.83 ± 17.93 fluorescence molecules per cluster. Fluorescence signals smaller than these sizes were eliminated as noises. An entire cell was optically sectioned in 50 nm z-steps (z-slices) with the range being between −500 and +500 nm that corresponds to between extracellular and intracellular depths. Then, 488/647 double positive objects that fulfilled intensity and area size of the Gb_3_ and Stx2 positive clusters in each z-slice were accordingly counted in order to quantify colocalization. Cells were visualized with 100 X magnification. Acquisition and analysis of 3D STORM-TIRF experiments were performed at The Institute of Human Virology Imaging facility at the University of Maryland School of Medicine.

### Live cell calcium dye imaging

Neuronal cultures at DIV7 were treated with a low affinity Ca^2+^-binding, cell-permeable dye fluo-5F- AM (ex494/em516 nm, 0.37 μM) in culture media for 30 min at 37°C and then incubated with fresh BSS buffer (1X Hank's balanced saline solution, 100 U/ml penicillin, 100 μg/ml streptomycin, 10 mM HEPES) for 3 h at 37°C. The neuron containing dish was placed on the confocal microscopy stage with a field having 3–5 slightly stained neuronal soma. Cells were visualized with argon laser (488 nm excitation) and the resting state of the fluorescence was recorded every 1 s for 1 min. Each cell body was circled as a region of interest (ROI) and the total intensity of a ROI for the first 1 min was averaged and defined as F0. Stx2 (3.45 μM in 2 μl volume) was added close to the imaging site after 1 min and the field was continuously recorded for a total recording time of 20 min. After Stx2 addition, the total intensity was defined as F, and this was normalized to F0. In experiments using inhibitors such as Wortmannin (final concentration 100 nM, EMD Millipore, Billerica, MA) or MK-801 (final concentration 20 μM, Sigma-Aldrich), these were added to the BSS buffer, or alternatively MK-801 was added simultaneously with Stx2 while imaging.

### Phosphorylated kinase array

Phospho-array assay (Proteome Profiler Antibody Array, R&D systems, Minneapolis, MN) was performed on neuronal lysates as per the manufacturer's instruction. Phospho-array is an antibody pre-spotted polyvinylidene fluoride (PVDF) membrane with 26 Phosphorylated-mitogen-activated protein kinases (MAPKs)-specific antibodies including p38, c-Jun N-terminal kinases (JNKs), protein kinase B (PKB/Akt), cAMP response element-binding protein (CREB), ribosomal s6 kinase (RSK), mechanistic target of rapamycin (mTOR), glycogen synthase kinase 3 beta (GSK3β) and extracellular-signal-regulated kinase (ERK)1/2. Neuronal cultures were treated with or without 1 nM Stx2 for 2 h at 37°C and lysates were made with RIPA buffer consisting 50 mM Tris, 150 mM NaCl, 1% igepal CA-630, 0.5% deoxycholic acid supplemented with protease inhibitor cocktail (all from Sigma-Aldrich), phenylmethanesulfonylfluoride (PMSF) (Roche, Nutley, NJ), orthovanadate and serine/threonine protein phosphatase inhibitor cocktail (EMD Millipore). Lysates were incubated with horseradish peroxidase-conjugated anti-phospho-antibody and applied to MAPK-specific antibody-bound PVDF membrane. ECL method was used to visualize positive signals (Amersham ECL Western blotting detection reagents, GE Healthcare Life Sciences, Pittsburgh, PA). The ECL reagent-treated phospho arrays were exposed to films which were scanned, and the chemiluminescent signals were quantified using ImagePro Plus (Media Cybernetics, Rockville, MD).

### TUNEL staining

We tested Stx2-treated cultured neurons for induction of apoptosis. Neurons were incubated with 0, 0.1, 1 or 10 nM of Stx2 for 48 h, and Terminal deoxynucleotidyl transferase dUTP Nick End Labeling (TUNEL) assay was performed as per the manufactures (EMD Millipore) instruction. DNase I (37 U/well, GE Healthcare Life Sciences) was used as a positive control, whereas media alone was used as negative control. TUNEL positive cells were counted with the aid of an Olympus BX40 (Olympus, Center Valley, PA) microscope using a magnification of 400x. Ten images per treatment were collected for statistical analysis, and TUNEL positive as well as total nuclei (methyl green positive) were counted per image.

We also performed TUNEL assays on CNS tissues from Stx2-injected mice for *in vivo* apoptotic evaluation. Mice were injected with 400 ng/kg Stx2 intraperitoneally and perfuse-fixed cerebral cortex and lumbar spinal cords were harvested immediately after injection (0 h) and at 2, 4, 6, 8, 12, 24, 48, or 60 h following injection. In addition, saline injected controls at 60 h, and normal (no injection) controls (*n* = 2 each) were performed. Tissues were processed and paraffin embedded. TUNEL was performed as noted above. The motor cortex and the ventral horn were visualized with 100 X magnification and TUNEL positive cells were counted (Two imaging fields (right and left)/section/mouse).

### PI/DAPI staining

Neuron cultures were treated with 0, 0.1, 1, and 10 nM Stx2 for 48 h at 37°C and further incubated with 0.5 μM propidium idodide (PI, Life Technologies) for 10 min at room temperature. After washing and fixing with 4% PFA/PBS, cells were counter stained with DAPI. Cells were observed with epifluorescence microscopy, Olympus BX40. Under 200x magnification in which approximately 100 cells can be captured, PI positive (red), and DAPI positive (blue) cells were counted. Ten images were counted in each treatment.

### Statistical analysis

Student's *t*-test was performed to compare control versus Stx2-treated group. Significance was noted for *p*-values less than 0.05 using two-tailed analysis. All statistical analyses were performed using GraphPad Prism software (GraphPad Software, Inc., La Jolla, CA).

## Results

### Stx2 interacts with neurons via surface expressed Gb_3_

**Figure 1:**
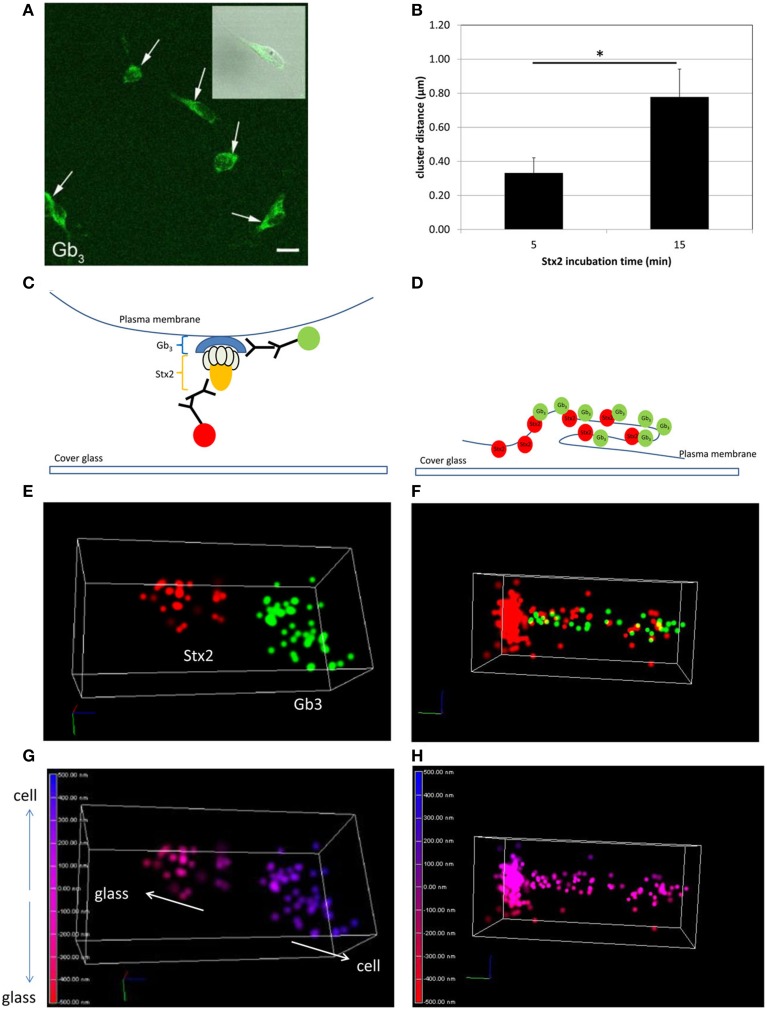
**Stx2 binds to cultured neurons via Gb_3_. (A)** Confocal microscopy images of anti-Gb_3_ Ab-AlexaFluor 488 (green, arrows) showing localization on the surface of neurons (DIV7). An inset shows merged image of Gb_3_ localization (green) and differential interference contrast (DIC). Bar indicates 10 μm. **(B)** Distance from the end of the green clusters to the beginning of the red clusters (*n* = 5 per group, ^*^p = 0.0007). **(C,E,G)** A representative STORM-TIRF image from 5 min Stx2-treated neurons. **(D,F,H)** A contact spot of Stx2 and Gb_3_ signals from 15 min Stx2 incubated neurons. **(C,D)** Depiction of indirect immunofluorescence of Gb_3_ [primary Ab against Gb_3_ and anti-rat Ab conjugated to AlexaFluor 488 (green)] and Stx2 [primary Ab against Stx2 and anti-mouse Ab conjugated to AlexaFluor 647 (red)] shown in cartoon format. **(E,F)** The STORM-TIRF images of contact spots from 5 min **(E)** and 15 min **(F)** incubations (Gb_3_ in green and Stx2 in red). **(G,H)** The depth conversions of **(E,F)**. The depth of the contact is color-coded from intracellular signal localization in blue (maximum depth of 500 nm) to extracellular localization in red (minimum depth of -500 nm) in continuous gradations where 0 nm (purple) indicates the focused plane (plasma membrane). An upward arrow in **(G)** shows intracellular direction (cell) and a downward arrow shows extracellular direction (glass).

In order to visualize direct Stx2-Gb_3_ interactions, we first conducted anti-Gb_3_ stain in neuronal culture to confirm Gb_3_ expression on the surface of cultured neurons. Neuronal cell bodies displayed Gb_3_ on the surface as confirmed by confocal microscopy (Figure 1A). In order to resolve Stx2-Gb_3_ interactions, we applied 3D STORM-TIRF, which allows better resolution compared to standard confocal microscopy (i.e., 3D STORM TIRF resolves 20 nm along the x- and y-axes and 50 nm along the z-axis versus standard confocal microscopy which is limited to 200 nm resolution). Following a 5 min incubation of neurons with Stx2, localization of Stx2 close to Gb_3_ was detectable (Figures 1E,G, a diagram in Figure 1C). Increasing the Stx2 incubation period to 15 min resulted in increased contacts of Stx2 and Gb_3_ signals which occurred in both clusters as well as in a tubular structure (Figure 1F, a diagram in Figure 1D). The tubular structures were formed intracellularly but close to the cell membrane (Figure 1H) suggesting endocytosis. The distance from the edge of the Stx2 signal to that of Gb_3_ was 0.324 ± 0.117 μm (*n* = 21) and 0.680 ± 0.139 μm (*n* = 5), for the 5 min and 15 min Stx2 incubations respectively (Figure 1B). The increased distance between Stx2 and Gb_3_ following the 15 min incubation period suggests a tubular invagination possibly associated with Stx2 uptake across the neuronal cell membrane.

### Stx2 induces glutamate release from the synaptic terminals of neurons

**Figure 2:**
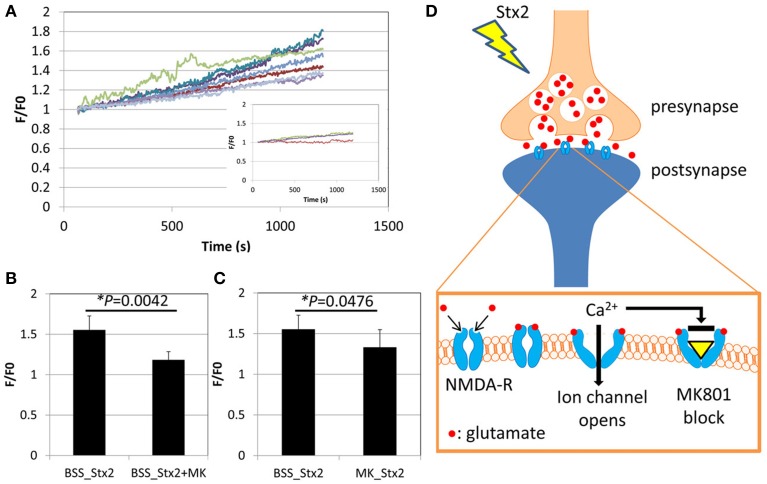
**Stx2 induces glutamate release from neurons**. Neurons were incubated with Ca^2+^ indicator dye FM5f-AM for 30 min at 37°C, 5% CO_2_, and incubated with BSS buffer with or without NMDA receptor antagonist MK-801 (MK) for 3 h. A confocal microscope LSM510 was used to visualize faintly stained cells with Argon laser (488 nm emission). **(A)** Live cell observation was started at 0 s, and Stx2 was added at 60 s and fluorescence was recorded until 1200 s. Neuronal cell bodies were determined as regions of interest. The baseline recordings from 0 to 60s were averaged and expressed as F0. For each recording point, fluorescence (F) was divided by F0 to calculate fold change (F/F0) and plotted. An inset shows traces of control neurons without Stx2. **(B)** Stx2 alone (*n* = 7) and Stx2 + MK (*n* = 4) added samples were compared their final F/F0 values at 1200 s. **(C)** Cells were incubated with (*n* = 9) or without (*n* = 7) MK and recorded as in **(A)** and analyzed as in **(B)**. In **(B,C)**, Student's *t*-test was performed. **(D)** A schematic presentation of Stx2-induced glutamate release and affected NMDA receptor. Glutamate is secreted from presynapse and binds to NMDA-receptors by which Ca^2+^ channels open, causing influx of detectable Ca^2+^. MK-801 binds to opened channel to block Ca^2+^ influx.

**Figure 3:**
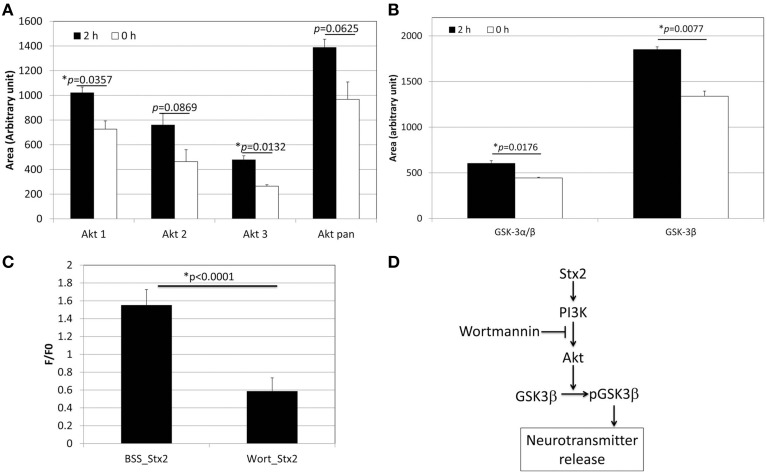
**Stx2 induces PI3K-involved signaling pathway to release glutamate. (A)** Phospho-array assay of neuronal lysates showed increased phosphorylation in Akt after 2 h incubation with 1 nM Stx2 (*n* = 2/probe). **(B)** GSK3β was also increased (*n* = 2/probe). **(A,B)** each depict a single example from a total of three samples. All experiments showed a similar trend. **(C)** Neurons were incubated with a PI3K inhibitor Wortmannin (Wort) and analyzed by Ca^2+^ imaging. Wortmannin significantly decreased Stx2-induced intracellular Ca^2+^ (BSS *n* = 7 and Wort *n* = 4, Student's *t*-test). **(D)** A schematic presentation of the proposed Stx2-induced PI3K/Akt/GSK3β pathway leading to neurotransmitter (glutamate) release.

Effects of Stx2 on neuronal intracellular Ca^2+^ was determined by visualization of a Ca^2+^ indicator dye in live cells. Neurons from postnatal day 0 (P0) mice were cultured until DIV7 and treated with Ca^2+^ indicator fluo-5F-AM. The cells were then imaged by confocal microscopy for a total of 20 min including baseline recording followed by Stx2 treatment. Individual neuronal cell bodies were determined as regions of interest (ROI) as we observed changes in fluorescent intensity in this region following Stx2 treatment. Each recorded Ca^2+^-bound fluo-5F-AM intensity value (F) was divided by baseline average (F0). Stx2 induced a gradual increase in F/F0 as shown in Figure 2A. An inset in Figure 2A shows F/F0 traces from a control experiment (without Stx2), and those traces appear much closer to zero than that from Stx2-treated cells. N-methyl-D-aspartate (NMDA) receptor is an ionotropic glutamate receptor which allows Ca^2+^ and Na^+^ to enter cells when bound by glutamate and glycine or D-serine. In order to test whether intracellular Ca^2+^ increase is due to activation of ionotropic NMDA receptors via Stx2-mediated glutamate release, we treated neurons with MK-801, which is a NMDA receptor-specific antagonist and can thereby block Ca^2+^ influx (Figure 2D). Neurons were either incubated with Stx2 and MK-801 simultaneously (BSS_Stx2 + MK) or pre-incubated with MK-801 before Stx2 addition (MK_Stx2). The final values of F/F0 were averaged and compared to Stx2 alone treatment (BSS_Stx2) (Figures 2B,C). When Stx2-treated samples (BSS_Stx2) were compared to samples with simultaneous treatment of Stx2 + MK-801 (BSS_Stx2 + MK), there was a significant reduction of F/F0 in Stx2 + MK-801 (*t*-test, ^*^p = 0.0042). Also, when cells were pre-incubated with MK-801 (MK_Stx2), Stx2-induced F/F0 was significantly reduced in MK-801 pre-treated cells (Figure 3C, ^*^p = 0.0476). The data suggests that the neurotransmitter released from Stx2-treated neurons is glutamate, resulting in postsynaptic NMDA-receptor activation and associated with Ca^2+^ influx.

### The PI3K signaling pathway is involved in Stx2-induced glutamate release in neurons

In order to determine the signaling pathway that Stx2 utilizes in neurons, a MAPkinase phospho-array was used to screen kinases that are phosphorylated in Stx2-treated neurons. Akt (subtypes Akt 1 and 3) and GSK3β were found phosphorylated after 2 h of Stx2 treatment (Figures 3A,B). Because Akt and GSK3β are known downstream molecules of the PI3K pathway, and GSK3β is associated with neurotransmitter release (Zhu et al., 2007), neurons were pre-incubated with a PI3K-specific inhibitor Wortmannin, and intracellular calcium was measured upon Stx2 addition. Wortmannin reduced F/F0 in Stx2-treated neurons suggesting the PI3K signaling pathway is involved in Stx2-induced glutamate release (Figure 3C).

### Stx2 induces neuronal death *in vitro* but not *in vivo*

**Figure 4:**
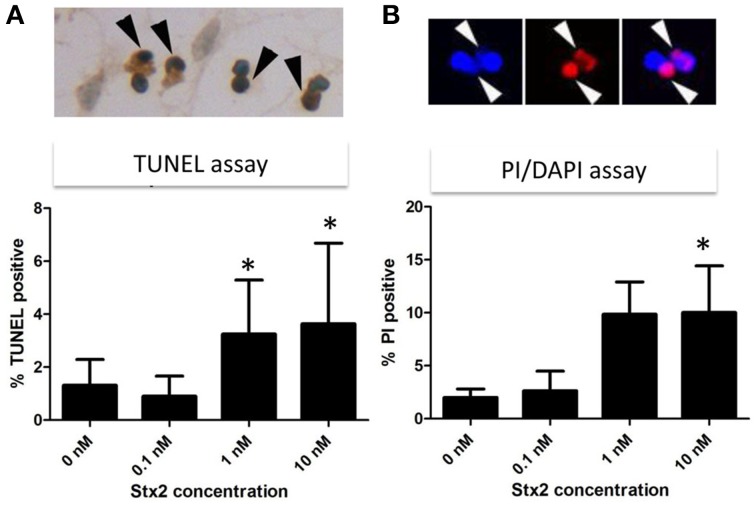
**Stx2 induces apoptotic cell death** ***in vitro***. Neurons were incubated with different concentrations of Stx2 for 48 h and assayed with TUNEL stain (*n* = 20/group) **(A)** and propidium idodide (PI)/DAPI stain (*n* = 5/group) **(B)**. TUNEL positive nuclei (dark brown, black arrowheads) numbers were normalized by counter stained total nuclei numbers and expressed as percent TUNEL positive. In PI/DAPI stain, the percentage of PI positive nuclei (Red, white arrowheads) per total (DAPI positive, blue) nuclei was calculated. One-Way ANOVA followed by Tukey test, ^*^p < 0.05.

High calcium influx induced by an excess glutamate is known to promote excitotoxicity by mechanisms including apoptosis (Yang et al., 2014). Also, we and others have shown in other cell types *in vivo* and *in vitro* that Stx2 can promote apoptosis (Kiyokawa et al., 1998; Jones et al., 2000; Kojio et al., 2000; Fujii et al., 2003, 2008; Wilson et al., 2005; Creydt et al., 2006; Stone et al., 2012; Dettmar et al., 2014), and therefore, we investigated *in vitro* and *in vivo*, whether Stx2 induced apoptosis in neurons. Stx2-induced apoptosis was measured by TUNEL stain. *In vitro*, TUNEL positivity was increased by Stx2 in a dose dependent manner suggesting Stx2 increases apoptotic cell death in neurons (Figure 4A), and PI/DAPI staining further confirmed these results (Figure 4B). While TUNEL assay detects DNA fragmentation that occurs during apoptotic cell death, the PI/DAPI assay is based on high impermeability of PI to nucleic membrane that PI nuclei stain only occurs when the membrane is irreversibly impaired (Wu et al., 2009), and does not distinguish between apoptosis and necrosis. Overall, both assays demonstrated that Stx2-treatment induced neuronal death *in vitro*. Contrarily, *in vivo* studies in mice where mice were administered 2LD_50_ dose of Stx2 intraperitoneally, we were unable to detect TUNEL positive neurons in the motor nuclei and the ventral horn of lumbar spinal cord (Table 1) that are responsible of moving hindlegs despite developing hindleg paralysis around 60 h of post injection, suggesting Stx2 does not induce apoptosis in murine neurons *in vivo*.

**Table 1:** **TUNEL results in spinal cord and cortex of Stx2-injected mice^a^**.

		**TUNEL (+) nuclei**			
		**Lumbar spinal cord^b^**		**Cerebral cortex^c^**	
**Stx2 (h)**	**Mouse ID**	**Neuron**	**Glia**	**Neuron**	**Glia**
0^d^	597	0	0	0	9
0	598	0	0	n.d.^e^	n.d.
2	599	0	0	n.d.	n.d.
2	600	0	0	n.d.	n.d.
4	601	0	0	n.d.	n.d.
4	602	0	0	n.d.	n.d.
6	603	0	0	n.d.	n.d.
6	604	0	0	n.d.	n.d.
8	605	0	0	n.d.	n.d.
8	606	0	0	n.d.	n.d.
12	607	0	0	n.d.	n.d.
12	608	0	0	n.d.	n.d.
24	609	0	0	n.d.	n.d.
24	610	0	0	0	5
48	611	0	2	n.d.	n.d.
48	612	0	1	n.d.	n.d.
60	613	0	0	0	5
60	614	0	0	n.d.	n.d.
saline 60^f^	615	0	0	0	9
saline 60	616	0	0	0	5
normal^g^	617	0	0	0	5
normal	618	0	0	n.d.	n.d.

a *One field of 200 X magnification per mouse was counted*.

b *Ventral horn including motor neurons*.

c *Motor nuclei*.

d *Stx2 (2LD50) was injected i.p. and sacrificed subsequently*.

e *N.d., not determined*.

f *Sacrificed after 60 h of saline injection*.

g *No injection control*.

## Discussion

Previously, we reported that mouse and human neurons express Gb_3_ *in vivo* (Obata et al., 2008). In the current study, we demonstrate for the first time that cultured murine neurons express Gb_3_ and that Stx2 interacts with Gb_3_ displayed on the surface of the cell. Moreover, by using 3D STORM-TIRF we are able to detect the development of an invaginating tubular structure following Stx2 treatment which was similar to that reported for the receptor binding subunit of Shiga toxin from Shigella dysenteriae type I in HeLa cells (Romer et al., 2007). These data suggest that Stx2 is able to intoxicate neuronal cells directly in a Gb_3_ dependent manner and that binding of Stx2 to Gb_3_ promotes structural changes in the cell membrane that may be indicative of early endocytosis.

Next we were able to show that murine neurons treated with Stx2 developed increased intracellular calcium. The occurrence of this gradual Ca^2+^ increase had us hypothesize that it could be triggered by Stx2-induced secondary factor such as glutamate release rather than direct ion channel opening by Stx2 To this extent we inhibited a subtype of ionotropic glutamate receptor, NMDA receptor with MK-801, which reduced Stx2-induced [Ca^2+^]_*i*_ increase, suggesting that synaptic glutamate release was contributing to the observed Stx2-mediated [Ca^2+^]_*i*_ increase. A previous study had shown that Shiga toxin type 1 induced PI3K/Akt signaling which was associated with Ca^2+^ signaling and phosphorylation of GSK3α/β in macrophage-like THP-1 cells (Cherla et al., 2009). Similar to those studies we observed that Stx2 caused increases in phospho-Akt isoforms and phospho-GSK3β (Figures 3A,B). The dephosphorylation of GSK3β has been demonstrated to reduce exocytosis of neurotransmitter in response to membrane depolarization in hippocampal neuronal culture (Zhu et al., 2010). Also, phosphorylation of GSK3β increases synaptic strength due to an increase in glutamate release (Hooper et al., 2007). To investigate the involvement of PI3K/Akt/GSK3β pathway in Stx2-induced glutamate release, we performed Ca^2+^ imaging assay using the PI3K inhibitor Wortmannin. Wortmannin-treated cells significantly reduced Stx2-induced [Ca^2+^]_*i*_ (Figure 3C, ^*^p < 0.0001). These data indicated that Stx2 induces glutamate release via the PI3K/Akt/GSK3β pathway. Usage of PI3K/Akt/GSK3β signaling pathway by Stx is in agreement with Cherla et al. (2009) in which Stx1 induced proinflammatory cytokine release from THP-1 cells.

*In vitro*, Stx2-treated neurons showed TUNEL positivity in dose dependent manner, and this trend was reproducible in PI/DAPI viability assay. This suggests that Stx2-induced death in neurons may be due to glutamate toxicity *in vitro*. However, *in vivo*, we did not observe TUNEL positivity in either neurons of motor nuclei in the cerebral cortex or motor neurons in the lumber spinal cord (Table 1). And the absence of neuronal death was reproducible in Nissl's stain which was used to evaluate general numbers of neurons (data not shown). *In vivo*, astrocytic end feet are usually surrounding synapses and contributing to scavenge presynaptically secreted excess glutamates via glutamate transporters to reduce excitotoxicity (Kawahara et al., 2002; Wisman et al., 2003). Thus, we speculate that it is possible *in vivo*, synapses may be initially protected by astrocytic glutamate scavenging function and Stx2-induced neuronal toxicity is avoided. However, during Stx2-induced hindlimb paralysis later in the time course, we observed that synapses surrounding motor neurons are interrupted by astrocytic end feet (Obata et al., 2008). This suggests association of astrocytes in Stx2-induced CNS dysfunction *in vivo*. Future studies of Stx2-affected neurons and astrocytes should help elucidate whether Stx2-induced glutamate release contributes to hindlimb paralysis and CNS impairment *in vivo*.

### Conflict of interest statement

The authors declare that the research was conducted in the absence of any commercial or financial relationships that could be construed as a potential conflict of interest.

## Supplementary material

Supplementary Video S1 **3D STORM-TIRF yellow cluster movie**. Green denotes Gb_3_ and red denotes Stx2.

Supplementary Video S2 **3D STORM-TIRF yellow cluster depth view movie**. The distribution of the molecules in Supplementary Video S1 is color coded according to the depth color bar.
